# Ecosystem service valuation and multi-scenario simulation in the Ebinur Lake Basin using a coupled GMOP-PLUS model

**DOI:** 10.1038/s41598-024-55763-6

**Published:** 2024-03-01

**Authors:** Hua Tang, Abudureheman Halike, Kaixuan Yao, Qianqian Wei, Lei Yao, Buweiayixiemu Tuheti, Jianmei Luo, Yuefan Duan

**Affiliations:** 1 College of Geography and Remote Sensing Sciences, Xinjiang University, Urumqi, 830017 China; 2 Xinjiang Key Laboratory of Oasis Ecology, Xinjiang University, Urumqi, 830017 China; 3 Key Laboratory of Smart City and Environment Modelling of Higher Education Institute, Xinjiang University, Urumqi, 830017 China

**Keywords:** Ecosystem service value, Land use and land cover change, GMOP-PLUS model, Multi-scenario simulation, Ebinur Lake basin, Biogeography, Ecological modelling, Ecosystem ecology, Ecosystem services

## Abstract

The Ebinur Lake Basin is an ecologically sensitive area in an arid region. Investigating its land use and land cover (LULC) change and assessing and predicting its ecosystem service value (ESV) are of great importance for the stability of the basin's socioeconomic development and sustainable development of its ecological environment. Based on LULC data from 1990, 2000, 2010, and 2020, we assessed the ESV of the Ebinur Lake Basin and coupled the grey multi-objective optimization model with the patch generation land use simulation model to predict ESV changes in 2035 under four scenarios: business-as-usual (BAU) development, rapid economic development (RED), ecological protection (ELP), and ecological–economic balance (EEB). The results show that from 1990 to 2020, the basin was dominated by grassland (51.23%) and unused land (27.6%), with a continuous decrease in unused land and an increase in cultivated land. In thirty years, the total ESV of the study area increased from 18.62 billion to 67.28 billion yuan, with regulation and support services being the dominant functions. By 2035, cultivated land increased while unused land decreased in all four scenarios compared with that in 2020. The total ESV in 2035 under the BAU, RED, ELP, and EEB scenarios was 68.83 billion, 64.47 billion, 67.99 billion, and 66.79 billion yuan, respectively. In the RED and EEB scenarios, ESV decreased by 2.81 billion and 0.49 billion yuan, respectively. In the BAU scenario, provisioning and regulation services increased by 6.05% and 2.93%, respectively. The ELP scenario, focusing on ecological and environmental protection, saw an increase in ESV for all services. This paper can assist policymakers in optimizing land use allocation and provide scientific support for the formulation of land use strategies and sustainable ecological and environmental development in the inland river basins of arid regions.

## Introduction

Ecosystem services refer to the natural environmental conditions and benefits provided and sustained by ecosystems and their operational processes for human society^1^. As a vital form of natural capital^2^, they are intimately linked with human well-being^3^. Ecosystem services encompass four main functions: provision, regulation, support, and cultural services^4–6^. However, with the ongoing developments in climate change and urbanization, they are facing significant challenges and pressures^7–9^. The International Geosphere-Biosphere Programme highlights that changes in the status, characteristics, and functions of ecosystems will inevitably impact the supply and demand balance and equitable distribution of ecosystem services^10–13^. This, in turn, can lead to increases or decreases in ecosystem service value (ESV). Quantitative analyses of ESV helps in gaining a more tangible understanding of the indispensable role ecosystems play in promoting economic growth, maintaining ecological balance, and ensuring ecological security^14^. This process not only aids decision-makers in developing more scientific and reasonable resource allocation and ecological management strategies but also provides a solid scientific foundation for the dual objectives of environmental protection and regional development^15^. Therefore, in the context of the ecological environment and land space planning systems, scientifically assessing ESV plays a crucial decision-making role.

The diversity and complexity of external environments pose a significant challenge to the accurate assessment of ESV^16^. Currently, such evaluations are not globally standardized^17^. In 1997, Costanza et al.^4^ introduced the equivalence factor method, a global assessment tool, which was later adapted for China in 2008 by Xie et al.^18^, resulting in a tailored set of ecosystem service equivalence factors for the country. Compared with the unit service function price approach, this method is advantageous due to its lower data requirements, simplicity in calculation, and higher degree of standardization^5,19^, and it has been widely adopted in domestic ESV assessments. However, this method represents the average level of ecosystem services across China. In smaller-scale assessments, the value coefficients must be adjusted based on the specific ecological background and spatial heterogeneity of the area^20^, creating tailored assessment models for different administrative units or watersheds.

Land use/land cover changes (LUCC) is crucial for evaluating ESV. LUCCs serve as the foundation for maintaining ecosystem stability and promoting sustainable development. It is also a critical indicator reflecting the impact of human activities on ecosystem disturbance, playing a vital role in supporting ecosystems^21,22^. The Future Earth program often focuses on the interplay between land use changes, ecosystem services, and human well-being at various levels^17^. With socioeconomic development, the excessive exploitation of land resources by humans has significantly damaged regional ecosystems^23^. LUCCs profoundly affect the structure, function, and spatial distribution of ecosystems, consequently altering their capacity to provide services^24,25^. This results in the loss of ESV in terms of regulation, supply, and culture^26^. Against this backdrop, balancing socioeconomic development with ecosystem protection and ensuring the continuous provision of ecosystem services pose global challenges^27,28^. Therefore, analyzing the spatiotemporal dynamics of LUCC and ESV and making informed predictions about future conditions are essential for enhancing ecosystem service functions and maintaining ecosystem health.

The development and refinement of LUCC simulation models have enabled a deeper exploration of the spatiotemporal evolution of land use and its driving mechanisms^29^. In recent decades, several academics have used complex spatial models, including Cellular Automata (CA)^30^, the CLUE-S model^31^, and Artificial Neural Networks (ANNs)^32^, to predict future LUCC and determine the response mechanisms of ESV to future LUCCs. The MOP model was integrated with the CLUE-S model to spatially allocate quantitative structure optimization results for ecological problems in Beijing^33^. Zhang et al.^34^ coupled the SD and FLUS models to establish various scenarios for land use simulation in the China–Pakistan Economic Zone. Many of these models cannot effectively optimize both the quantity and spatial features of LUCC simultaneously^35,36^. Liang et al.^37^ developed a Patch Generation Land Use Simulation (PLUS) model based on multi-type random patch seeds that enhances the traditional CA framework by integrating mixed cells, which reflect the complexity of real-world land structures^38^. This model incorporates a Land Expansion Analysis Strategy (LEAS) with the CA model, facilitating a shift from qualitative, static simulations to quantitative, dynamic ones^39,40^. Compared with the models mentioned above, the PLUS model has strong reliability and robustness, and it exhibits landscape pattern indicators that closely resemble those observed in real landscapes^41^. Traditional land use planning methodologies often base future development objectives on historical trends in LUCCs^42^. However, this approach to forecasting falls short in effectively fostering the sustainable development of ecosystems. Currently, the prediction of LULC and ESV using coupled models such as the PLUS model, MOP model, and SD model is gradually becoming a research hotspot^39^. The coupled model can more clearly show the complexity of LUCC and its impact on ESV and provide decision-makers with advice on future LULC patterns.

In light of the pronounced dichotomy between urbanization and ecological conservation, existing land use planning frameworks must be refined. This entails pioneering diverse development trajectories to guarantee the harmonious and integrated progression of LUCC across the three critical sectors of production, living, and ecology^43^. To this end, this study couples the MOP and PLUS models, utilizing the two models in macro-level land demand modeling and micro-level land allocation. The study also combines the GM (1,1) grey linear prediction model to determine the ideal land use schemes in the Ebinur Lake Basin under four scenarios: business-as-usual (BAU) development, rapid economic development (RED), ecological protection (ELP), and ecological–economic balance (EEB). The aim is to explore LUCC development strategies under different scenarios that prioritize economic growth, environmental protection, or a balance of both. This approach is intended to provide more scientific and effective support and guidance for future land use planning.

We selected the Ebinur Lake Basin, located in the arid northwest region of China, as our study area. As a quintessential example of a lake basin in an arid zone, the Ebinur Lake Basin is characterized by its fragile ecological environment and diverse landscape types, integrating wetlands and desertification processes^44^. It stands as a crucial area for biodiversity conservation and natural ecological functions. However, as urbanization intensifies and the tourism industry expands, the tension between human activity and the land sharply escalates^12^, leading to increasingly pronounced disturbances to the Ebinur Lake Basin ecosystem. Therefore, evaluating and predicting the spatial variation in ESV against the backdrop of the Basin's LUCC is critical^45^. Such analysis is vital not only for preserving the ecological stability of the Ebinur Lake Basin but also for steering it towards a path of sustainable development. The purpose of this study is to provide well-informed decision-making references for the management and land use optimization projects in the Ebinur Lake Basin and offer a scientific foundation for the ecological environmental security and sustainable development of ecosystems in arid region basins.

## Material and methods

### Study area

**Figure 1:**
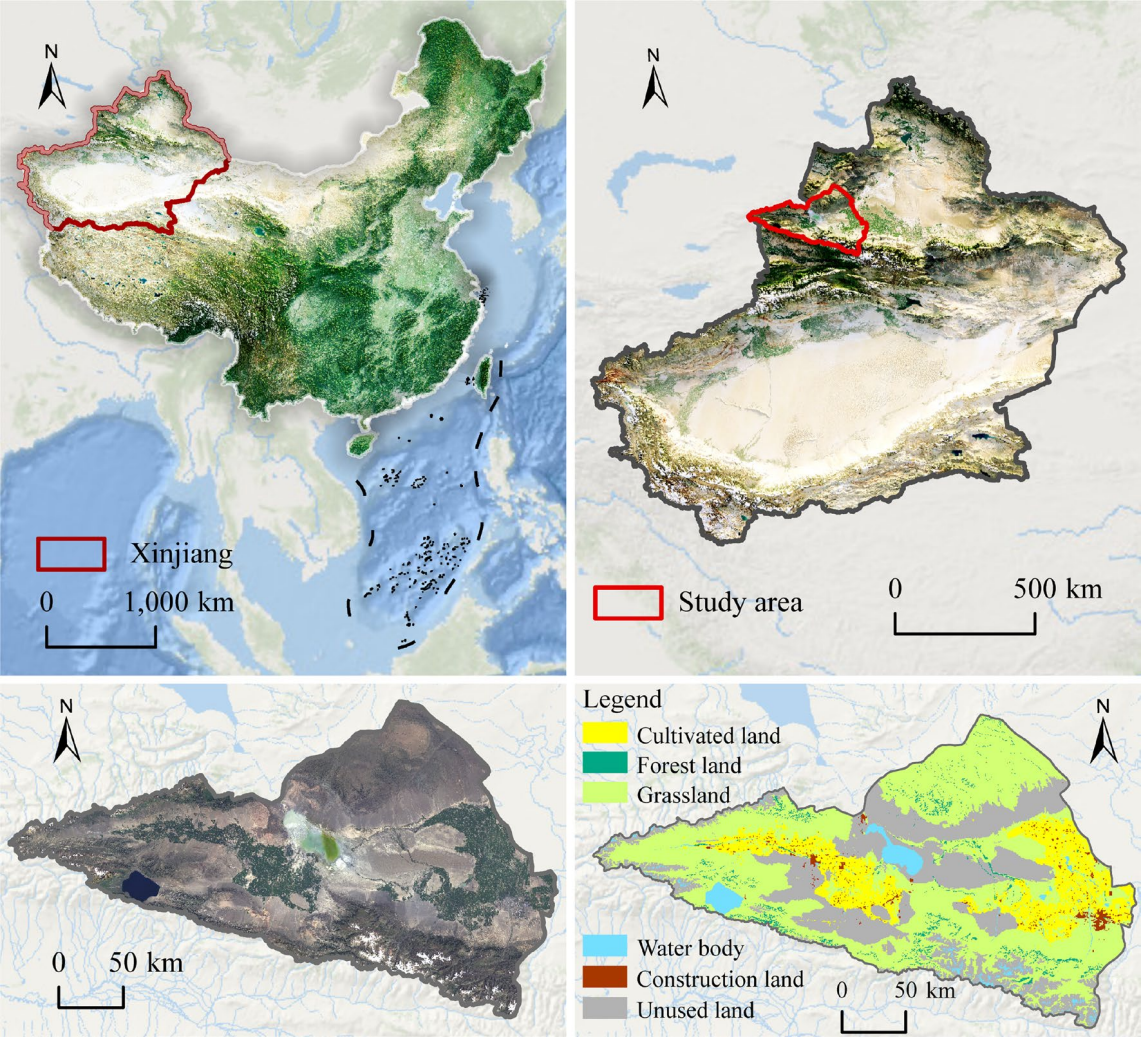
Location of the Ebinur Lake Basin (Software: ArcMap 10.2.0, http://www.esri.com).

The Ebinur Lake Basin is situated in the northwest of Xinjiang (43° 38′–45° 52′ N, 79° 89′–85° 38′ E; Fig. 1), an autonomous region of China. Its total area is 5,035,616 hm^2^, and it has a temperate continental climate. This area is arid with low rainfall and strong evaporation; the average annual precipitation ranges from 100 to 200 mm, and annual evaporation from 1500 to 2000 mm^46^. Lake Ebinur, as a typical arid zone lake, features a unique wetland desert ecosystem. The land use within its basin primarily consists of grasslands (51%) and unused lands (28%)^47^. Currently, land use within the Lake Ebinur Basin shows an increasing trend, with water conservation, soil formation and protection, and waste treatment being its main ecosystem service functions^48^. Although the expansion of land use types with high ecological value can improve the overall ecological quality of the basin, the entire ecological environment remains relatively fragile.

Since the Pleistocene, Lake Ebinur has been undergoing a natural process of desiccation and shrinkage^49^, leading to a severe decline in the surrounding natural vegetation. This not only restricts the development of agriculture and animal husbandry but also exacerbates regional sand and dust weather due to the exposure of the lakebed rock layers^16^. In recent years, the intensification of industrial and agricultural production activities has also led to numerous ecological and environmental problems in the basin, including soil erosion, forest destruction, and soil salinization.

### Data sources and pre-processing

This study used land use type, a digital elevation model, climate, normalized difference vegetation index (NDVI), net primary productivity (NPP), and socioeconomic data. Details of the data sources, years, and resolutions are listed in Table 1. To maintain consistency in spatial data accuracy, all data were resampled at a resolution of 100 m.

**Table 1:** Details of the dataset and data source information used in this study.

Type	Data	Year	Resolution	Source
Land dataset	Land use/land cover	1990–2020	30 m	https://www.resdc.cn/
Terrain	Elevation slope aspect	2010	30 m	https://www.nasa.gov
Meteorological data	Precipitation temperature	1980–2020	1 km	http://data.tpdc.ac.cn
	Dryness	1971–2000	1 km	http://www.nesdc.org.cn/
Vegetation	Normalized difference vegetation index	1990–2020	30 m	https://developers.google.com/earth-engine/
	Net primary productivity	1990–2020	30 m	https://developers.google.com/earth-engine/
Soil data	Soil types	2000	1 km	http://vdb3.soil.csdb.cn
	Soil erosion	1995	1 km	https://www.resdc.cn/
Spatial accessibility	Distance to residents	2021	100 m	https://www.ngcc.cn
	Distance to government			
	Distance to road			
	Distance to lake and river			
Socioeconomic factors	Gross domestic product population	2019	1 km	https://www.resdc.cn/
	Nighttime light data	2020	500 m	https://www.ngdc.noaa.gov/
	Crop area and yield	1990–2020		China Statistical Yearbook, Xinjiang Statistical Yearbook, Bortala Mongol Autonomous Prefecture Statistical Yearbook

### Research framework

**Figure 2:**
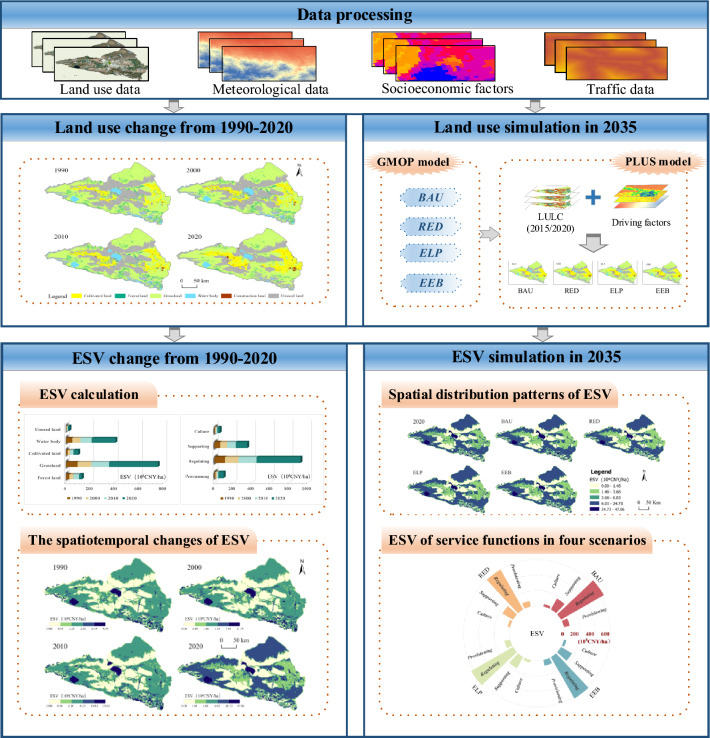
Research framework of this study (Software: ArcMap 10.2.0, http://www.esri.com. OriginPro 2022 SR1, https://www.originlab.com/. Visio 2021, https://visio.iruanhui.cn/).

The research method included four steps (Fig. 2): (1) characterization of LUCC in the Ebinur Lake Basin from 1990 to 2020; (2) identification of the spatiotemporal evolution pattern of ESV based on LUCC; (3) coupling of the Grey Multi-objective Optimization (GMOP)-PLUS model to simulate and optimize LUCC in the Ebinur Lake Basin for 2035; and (4) simulation of ESV under four scenarios in 2035 and analysis of their spatial evolution patterns.

### GMOP algorithm: Optimization of land use structure

A Multi-Objective Programming (MOP) model was employed to investigate the optimization of multiple objective functions within the specified region. The incompleteness of land use system information belongs to the grey system. The GMOP is derived from the GM (1,1)^50^ grey linear prediction and MOP models and can flexibly incorporate various ecological or macroeconomic policies^51,52^. By constructing suitable goal functions and constraints and identifying the optimal solution, the GMOP can be used to optimize LULC.

#### Scenario setting

Given the inherent difficulties in quantifying social benefits, this study constructs three optimization objectives based on economic and ecological benefits and combines them with the GM (1,1) model:

(1) $max\{{f}_{1}({\text{x}})\}$, Maximize economic benefits. The function can be expressed as:

1 $${f}_{1}\left({\text{x}}\right)=\sum_{i=1}^{6}{E}_{i}\cdot {x}_{i}$$

where ${E}_{i}$ represents the economic benefit of the $i$-th land use type per unit area, and ${x}_{i}$ represents the area of the $i$-th land use type. The economic benefit objective function of the Ebinur Lake Basin is therefore:

2 $${f}_{1}\left({\text{x}}\right)=4.5{x}_{1}+2.37{x}_{2}+1.02{x}_{3}+0.02{x}_{4}+443.72{x}_{5}+0{x}_{6}$$

$(2) max\{{f}_{2}({\text{x}})\}$, Maximize the ESV. The function can be expressed as:

3 $${f}_{2}\left({\text{x}}\right)=\sum_{i=1}^{6}{V}_{i}\cdot {x}_{i}$$

where ${V}_{i}$ represents the ESV of the $i$-th land use type per unit area. The final objective function is as follows:

4 $${f}_{2}\left({\text{x}}\right)=0.64{x}_{1}+2.88{x}_{2}+1.93{x}_{3}+14.6{x}_{4}+0{x}_{5}+0.12{x}_{6}$$

(3) $max\{{f}_{3}({\text{x}})\}$, Maximizing ecological capacity. The objective function can be expressed as:

5 $${f}_{3}\left({\text{x}}\right)=\sum_{i=1}^{6}{C}_{i}\cdot {x}_{i}\times \left(100-12\right)\mathrm{\%}$$

where ${C}_{i}$ represents the ecological capacity of the *i-*th land use type per unit area. The value of ${C}_{i}$ is obtained by multiplying the yield and equilibrium factors^53,54^. When calculating ecological capacity, 12% of the biological production land area should be deducted for biodiversity protection^55^. Therefore, the objective function is:

6 $${f}_{3}\left({\text{x}}\right)=4.64{x}_{1}+0.59{x}_{2}+0.50{x}_{3}+0.18{x}_{4}+4.64{x}_{5}+0{x}_{6}$$

In addition, four scenarios were established to project the Ebinur Lake Basin LUCC in 2035: (1) BAU: obtained through Markov chain prediction; (2) RED: maximizing economic benefits using the objective function $max\left\{{f}_{1}\left({\text{x}}\right)\right\}$; (3) ELP: maximizing ecological benefits using the objective function $max\left\{{f}_{2}\left({\text{x}}\right),{f}_{3}\left({\text{x}}\right)\right\}$; and (4) EEB: $max\{{f}_{1}\left({\text{x}}\right),{f}_{2}\left({\text{x}}\right),{f}_{3}({\text{x}})\}$, balancing economic advancement and ecological preservation to achieve sustainable development.

#### Constraint conditions

(1) Total area:

The aggregate of the areas corresponding to each land use category was equivalent to the overall extent of the Ebinur Lake Basin.

7 $$\sum_{i=1}^{6}{x}_{i}=5035616 \,{{\text{hm}}}^{2}$$

(2) Population:

The population of agricultural and urban land in the study area was limited to the predicted population of the target year.

8 $$\otimes \left( {a_{21} } \right)\left( {\mathop \sum \limits_{i = 1}^{3} x_{i} } \right) + \otimes \left( {a_{22} } \right)\left( {x_{5} } \right) \le P$$

where $P$ is the projected population size for 2035. According to historical statistical data and using the GM (1,1) model, we get: $\otimes ({a}_{21})\in (\mathrm{0.07,0.05})$, $\otimes ({a}_{22})\in (\mathrm{4.31,6.73})$, $P$ = 563,578.

(3) Food demand:

Cultivated land meets the demand of the study area to secure a food supply.

9 $${x}_{1}\cdot {f}_{1}\cdot {f}_{2}\cdot {f}_{3}\ge P\cdot S\cdot {f}_{0}$$

where $S$ is the per-capita food requirement, ${f}_{0}$ is the food self-sufficiency rate, ${f}_{1}$ is the grain yield per unit area, ${f}_{2}$ is the crop cultivation ratio, and ${f}_{3}$ is the multiple-cropping index. Based on previous research^56^ and the GM (1,1) prediction, we obtained $S=538\mathrm{ kg}$, ${f}_{0}=1$, ${f}_{1}=\mathrm{10,845 kg}/{\text{ha}}$, ${f}_{2}=26.68\mathrm{\%}$, ${f}_{3}=1.29$.

In addition, according to the growth rate in the past 30 years, the cropland in 2035 will be 10% lower than the Markov chain predicted value increase; that is, the upper limit is 957,862 hm^2^.

(4) Forest cover:

Liu et al.^57^ introduced the “green equivalent” to calculate forest cover. The coefficients for the three land types that meet the green equivalent are set at 0.46, 1.00, and 0.49, respectively. The United Nations stipulates that the lower limit of average forest cover should not be less than 20%. Therefore, we considered 20% of the total study region as the lower limit of forest cover. The formula was expressed as:

10 $$0.46{x}_{1}+{x}_{2}+0.49{x}_{3}\ge 5035616\times 20\mathrm{\%}$$

Simultaneously, according to the reduction trend, we considered the prediction of 92,537 hm^2^ as the lower limit. Because the government will implement more forest protection policies, forest cover will be higher than it currently is. Thus, the forest area in 2015 was established as the maximum limit, and the constraint condition can be written as:

11 $$92537\le {x}_{2}\le 121697 \,{{\text{hm}}}^{2}$$

(5) Grassland:

The grassland in the study region has exhibited an upward trend over the last 20 years but a declining trend within the most recent five-year period. The upper and lower bounds of the grassland areas were set as the maximum and minimum values for 2010–2020, and the formula was as follows:

12 $$2364064\le {x}_{3}\le 2616499 \,{{\text{hm}}}^{2}$$

(6) Water body:

According to the changing trend of the rise and fall of the water area in the study region during different periods over the past 20 years, together with global warming and intensified evaporation, the water area may decrease further in the future. Consequently, the maximum limit for the water body was set using the values for 2020, whereas the lower limit was defined using those from 2015. The formula was expressed as:

13 $$148363\le {x}_{4}\le 166058{ \,{\text{hm}}}^{2}$$

(7) Construction land:

Based on the overall goal of the “General Plan for Land and Space of Bortala Mongolian Autonomous Prefecture (2021–2035),” a “one group, two belts, and one axis” urban spatial structure will be created in the future, which will not be lower than the construction land in 2015, and the future rise in construction land area will be controlled within 50% of the area in 2020. The constraint condition can be written as:

14 $$62739\le {x}_{5}\le 91707 \,{{\text{hm}}}^{2}$$

(8) Unused land:

In recent years, the government has implemented comprehensive ecological and environmental remediation projects and the area of unused land has continued to decline. Therefore, we took the prediction of 1,326,661 hm^2^ as the maximum limit for 2035. The formula was expressed as:

15 $$0\le {x}_{6}\le 1326661{ \,{\text{hm}}}^{2}$$

(9) Model:

In the model, each constraint variable must meet the following conditions:

16 $$x_{i} \ge 0,i = 1,2,3 \ldots 6$$

where $i$ represents the $i$-th land use type.

### PLUS model: land-use spatial configuration

The PLUS model^58^ is a CA model based on the LEAS rule-mining framework and multitype random seed model. A comparative analysis with other models, namely CLUE-S and CA-Markov, revealed that the PLUS model exhibited superior credibility and more similar landscapes that closely resembled the observed patterns^37^.

#### Driving factors selection

Considering the unique natural geographical features and socioeconomic progress in the study region, we selected 18 driving factors in total (Table 1).

#### Model accuracy validation

We used two methods to verify the model accuracy. The first was by calculating the kappa coefficient and overall accuracy. Previous studies have shown that a kappa coefficient value greater than 0.8 indicates a high level of accuracy in model simulations^59^. The kappa coefficient in this study was 0.94, and the overall accuracy was 96.63%, indicating that the model has excellent credibility. The second method involved determining the coefficient of the figure of merit (FOM), in which a higher value indicates greater accuracy in the simulation findings. The FOM coefficients range between 1 and 59%, with a median value generally between 0.01 and 0.25^60^, and previous studies have shown that the FOM of urban land use dynamic simulation is between 12 and 18%^60,61^. Therefore, the FOM of 18.17% in this study indicates that the PLUS model accuracy is within acceptable limits.

### Ecosystem service valuation

In 2001, Xie et al.^62^ introduced the ESV valuation table formulated by Costanza et al.^4^ to China, and then established an equivalent ESV table suitable for China, which has subsequently been revised twice. The formula for determining the ESV can be expressed as:

17 $$V{C}_{i}=\sum_{f=1}^{k}{E}_{a}\times E{C}_{f}$$

18 $$ESV=\sum_{i=1}^{n}{A}_{i}\times V{C}_{i}$$

where $V{C}_{i}$ is the basic equivalent of the *i-*th land-use type, ${E}_{a}$ represents the standard equivalent, $E{C}_{f}$ is the equivalent coefficient, and ${A}_{i}$ is the acreage of the $i$-th land category.

#### Equivalent correction of ESV

Owing to spatial heterogeneity and regional economic development imbalance, the revision of the equivalent table includes the following^62^:

(1) Determination of the standard equivalent of ESV.

We selected the main grain crops (corn and wheat) in the study region and calculated the values of the standard equivalents in 1990, 2000, 2010, and 2020 as 2006, 2043, 2107, and 2181 yuan/hm^2^, respectively.

(2) Revision of the unit-area ESV equivalent coefficient table.

This study selected NPP and fraction of vegetation cover (FVC) to spatially and heterogeneously revise the ESV coefficient, as shown in the following formula:

19 $${Q}=(\frac{NP{P}_{i}}{NP{P}_{\text{j }}}+\frac{FV{C}_{i}}{FV{C}_{\text{j }}})/2$$

where $NP{P}_{i}$ and $NP{P}_{\text{j}}$ are the mean NPP values of the study region and nation as a whole, respectively, and $FV{C}_{i}$ and $FV{C}_{\text{j}}$ are the mean FVC values of the study region and nation as a whole, respectively.

This study revised the social development coefficient from two aspects; payment ability and willingness-to-pay, as shown in the following formula:

20 $$PI=A\times W$$

where $PI$ is the social development correction coefficient, $A$ represents payment ability, and $W$ represents willingness-to-pay.

21 $$A=\frac{{GDP}_{i}}{{GDP}_{j}},W=\frac{{L}_{i}}{{L}_{j}}$$

where ${GDP}_{i}$ and ${GDP}_{j}$ represent the per capita gross domestic product of the study region and nation as a whole, respectively, and ${L}_{i}$ and ${L}_{j}$ represent the social development stage coefficients of the study region and nation as a whole, respectively.

22 $$L = \frac{1}{{1 + e^{{ - (1/E_{n} - 3)}} }}$$

where $e$ denotes the natural constant, and ${E}_{n}$ represents the Engel coefficient.

## Results

### Land use evolution from 1990 to 2020

**Figure 3:**
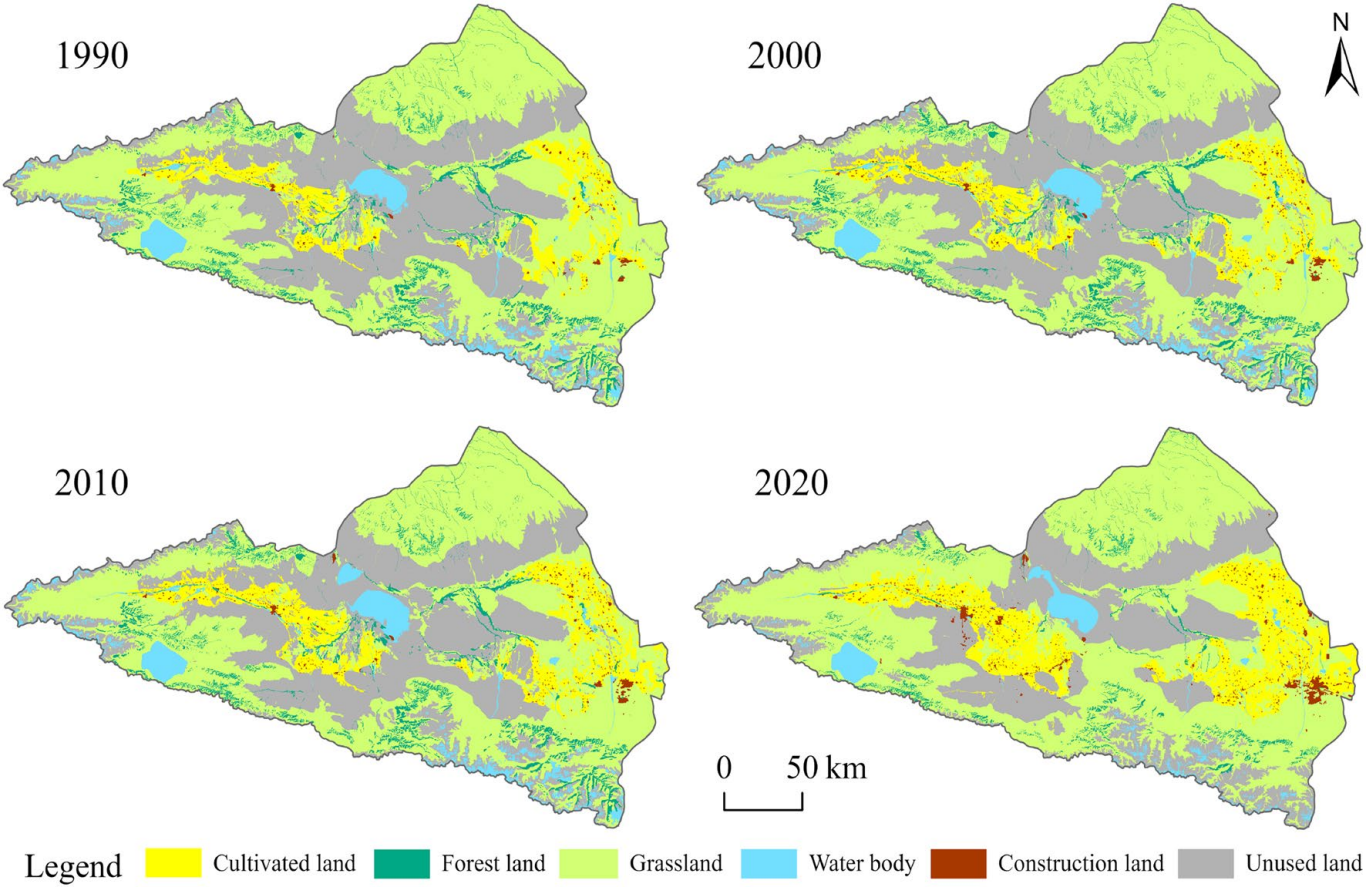
Spatial distribution of land use/land cover change (LUCC) from 1990 to 2020 in the study area (Software: ArcMap 10.2.0, http://www.esri.com).

**Figure 4:**
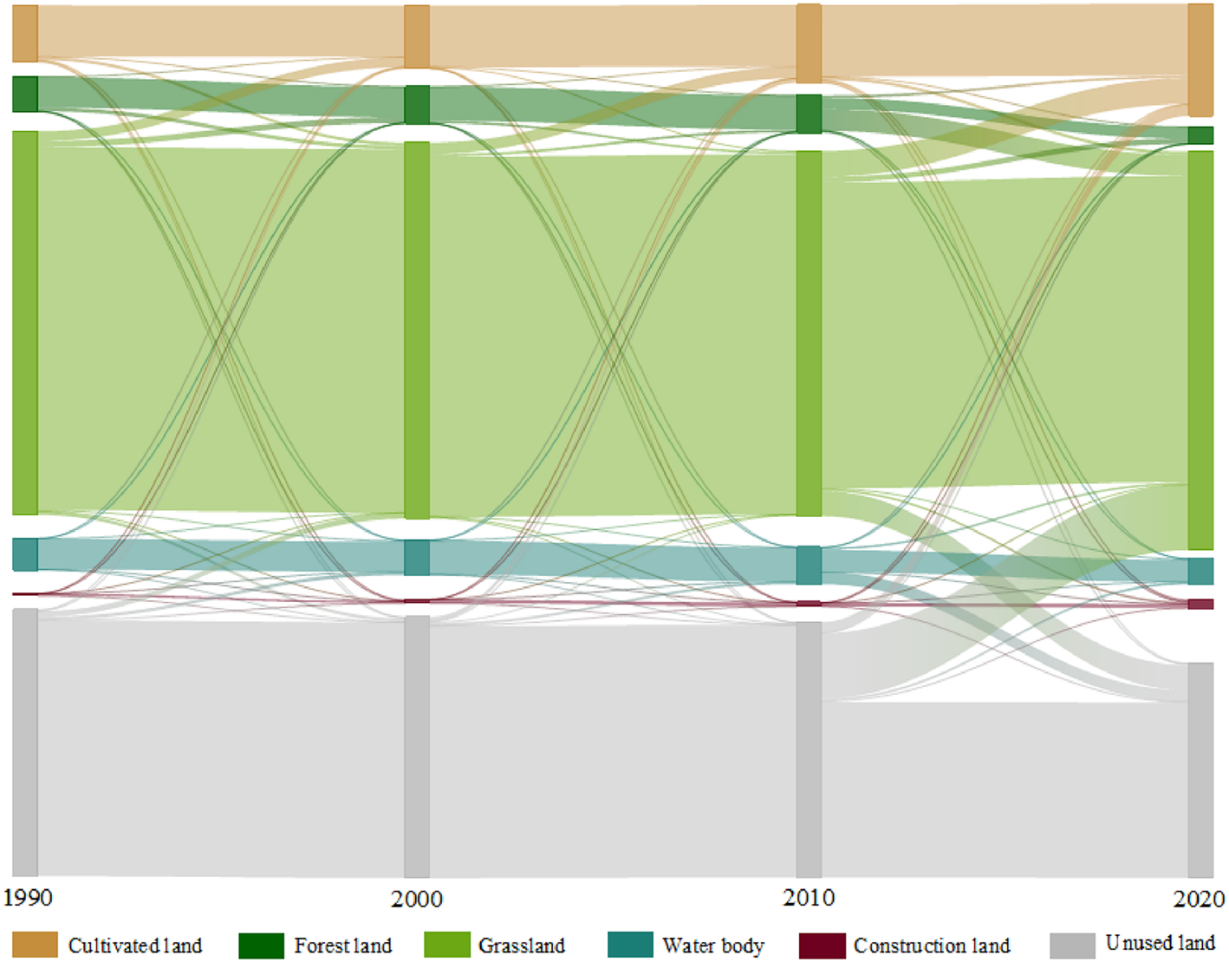
Sankey diagram of LUCC from 1990 to 2020.

In the study area, the main land use types are grassland and unused land (Fig. 3), accounting for 51.23% and 27.6% of the total watershed area in 2020, respectively. From 1990 to 2020, the areas of cultivated land and construction land steadily increased, while grassland area first decreased then increased, and unused land continuous decreased, mainly transforming into grassland and cultivated land. Forests and water bodies exhibited a fluctuating decrease, with the water area peaking in 2010 at 240,585 hm^2^. Due to climate warming, ice and snow coverage in the southeastern mountainous area of the study region significantly reduced, leading to a decrease in water area to 166,058 hm^2^ in 2020. The transitions of various land use types are illustrated in the Sankey diagram, Fig. 4. Due to urbanization and population growth, which increased the demand for food, the area of cultivated land nearly doubled from 369,621 hm^2^ in 1990 to 725,534 hm^2^ in 2020 (Table 2). Construction land, mainly distributed around cultivated areas, has grown over the past 30 years from 16,232 to 61,138 hm^2^, with the highest dynamism rate among the six land use types at 9.22%. With the development of socioeconomics and urbanization, construction land is expected to expand further in the future.

**Table 2:** Area and dynamic degree (K) of various land use/land cover (LULC) types from 1990 to 2020.

LULC type		Cultivated land	Forest land	Grassland	Water body	Construction land	Unused land
1990	Area (hm^2^)	369,621	228,611	2,484,147	205,497	16,232	1,729,175
	%	7.34	4.54	49.35	4.08	0.32	34.35
2000	Area (hm^2^)	409,343	242,211	2,435,748	225,707	25,689	1,692,813
	%	8.14	4.81	48.41	4.49	0.51	33.64
2010	Area (hm^2^)	504,667	241,860	2,364,064	240,585	29,468	1,653,245
	%	10.03	4.80	46.96	4.78	0.59	32.84
2020	Area (hm^2^)	725,534	113,439	2,579,527	166,058	61,138	1,389,920
	%	14.41	2.25	51.23	3.30	1.21	27.60
1990–2020	K (%)	3.21	− 1.68	0.13	− 0.64	9.22	− 0.65

### Spatiotemporal characteristics of ESV

**Figure 5:**
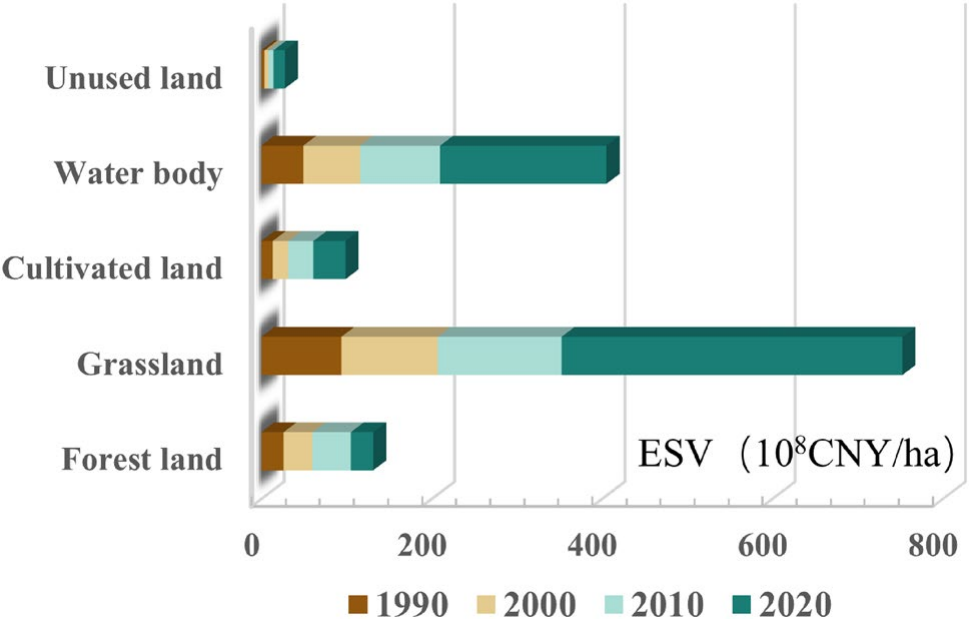
ESV of each land use type from 1990 to 2020.

We calculated the ESV for each land use type and the value of individual ecosystem service functions (Fig. 5) based on the area of various LULC types (Table 2) and modified value equivalent coefficients for each study year (1990, 2000, 2010, 2020). The results indicate that from 1990 to 2020, the total ESV of the study area showed a continuous upward trend, increasing from 18.62 billion to 67.28 billion yuan, with a cumulative increase of 48.66 billion yuan. The land use types contributing the most to the total ESV in the study area were grasslands and water bodies. In 1990, they collectively accounted for 76.82% of the total, rising to 88.52% by 2020, owing to the largest coverage area of grasslands and the highest ecological value coefficient of water bodies. Due to the very low ecological value coefficient of unused land, it only accounted for 1.84–2.04% of the total ESV within the assessment years, despite occupying 34.35–27.6% of the total area of the study region. Based on the ESV growth trend over the years, grasslands and water bodies still made the highest contribution to the increase in ESV, increasing by 30.64 billion and 14.61 billion yuan from 1990 to 2020, respectively, and collectively accounting for 93% of the total ESV increase. The growth in population led to an increased demand for farmland, causing a continuous increase in its contribution to the ESV, from 1.33 billion yuan in 1990 to 3.76 billion yuan in 2020. Although forests have a high ecological value coefficient, their area decreased over the study period, contributing only 0.05% to the ESV increase.

**Figure 6:**
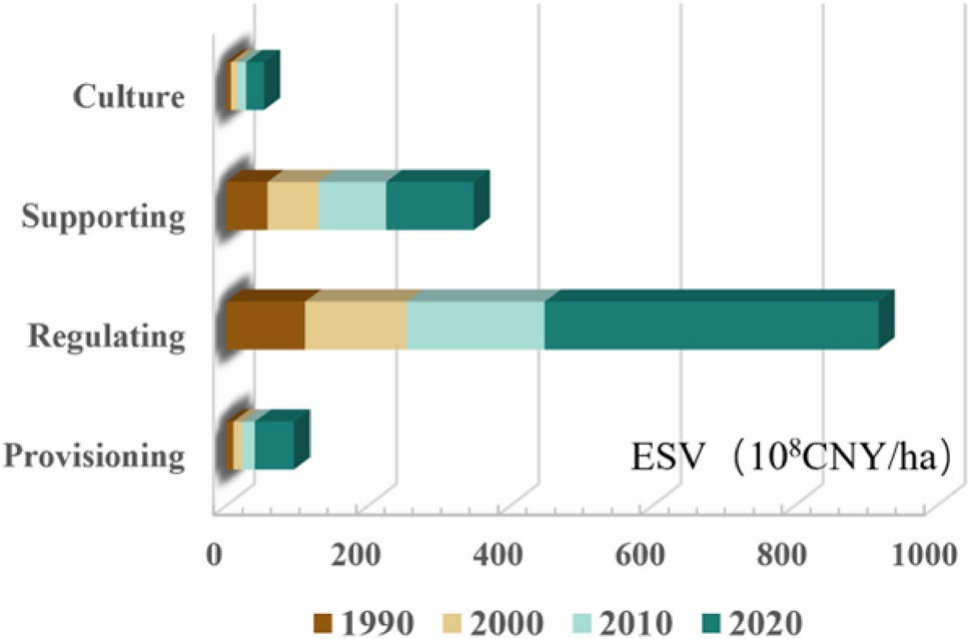
ESV of each service function from 1990 to 2020.

Furthermore, we calculated the values of individual ecosystem service functions and evaluated the ESV of four types of services: provisioning, regulation, support, and culture (Fig. 6). Their ESVs generally showed an increasing trend, but the differences in the increments between the functions were evident. From 1990 to 2020, regulation services showed the largest increase, accounting for 73.63% of the total increase, whereas provisioning and cultural services showed a slight upward trend. The primary service functions in the study region were regulation and support services, which accounted for approximately 90% of the overall ESV, whereas cultural services accounted for only 3.7%. Among these, water conservation and waste treatment within the regulatory services had the greatest impact on the ESV, primarily due to the strong water conservation and waste treatment capabilities of aquatic ecosystems, coupled with the high value coefficients of their ecosystem service functions. Following these are the supporting services of soil conservation and biodiversity protection, which together account for approximately 30% of the total ESV. However, because water bodies occupy a smaller proportion in the study area and grasslands are the predominant cover type, the incremental value of individual ecosystem service functions in the entire study area is primarily driven by grasslands and aquatic ecosystems.

**Figure 7:**
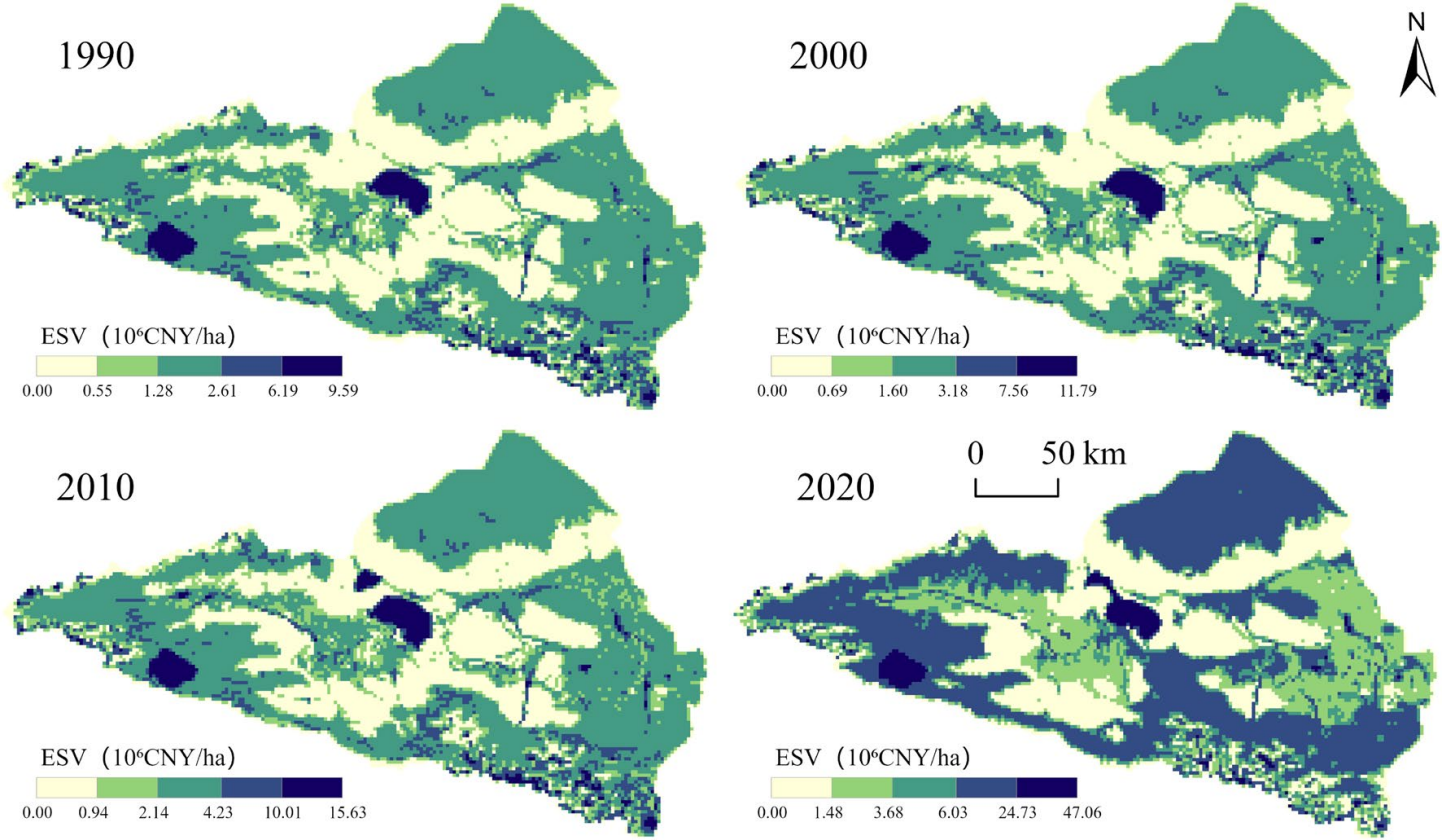
ESV spatial distribution maps from 1990 to 2020 (Software: ArcMap 10.2.0, http://www.esri.com).

The spatial distribution of regional ESV is closely related to the spatial pattern of LULC. In this study, the natural breakpoint method is used to categorize the ESV into five levels, as shown in Fig. 7, to assess its spatial distribution in the study area from 1990 to 2020. The results indicate that, over the past 30 years, the ESV distribution pattern has generally remained stable but exhibits significant spatial heterogeneity. The high-value zone primarily consists of water bodies, including two large lakes, Lake Ebinur and Lake Sayram, and glaciers in the southeast. The mid-value areas are spread over most of the study area and are largely distributed in grassland and forest ecosystems with less human disturbance. The low-value zone is located on unused land and is concentrated, contiguous, and scattered on construction land characterized by rapid population growth. Most areas show a continuous upward trend in ESV, whereas the declining areas are point-like and distributed in the southeast, mainly because of global warming causing glaciers to melt and turn into unused land.

### LUCC in 2035 by GMOP-PLUS

**Figure 8:**
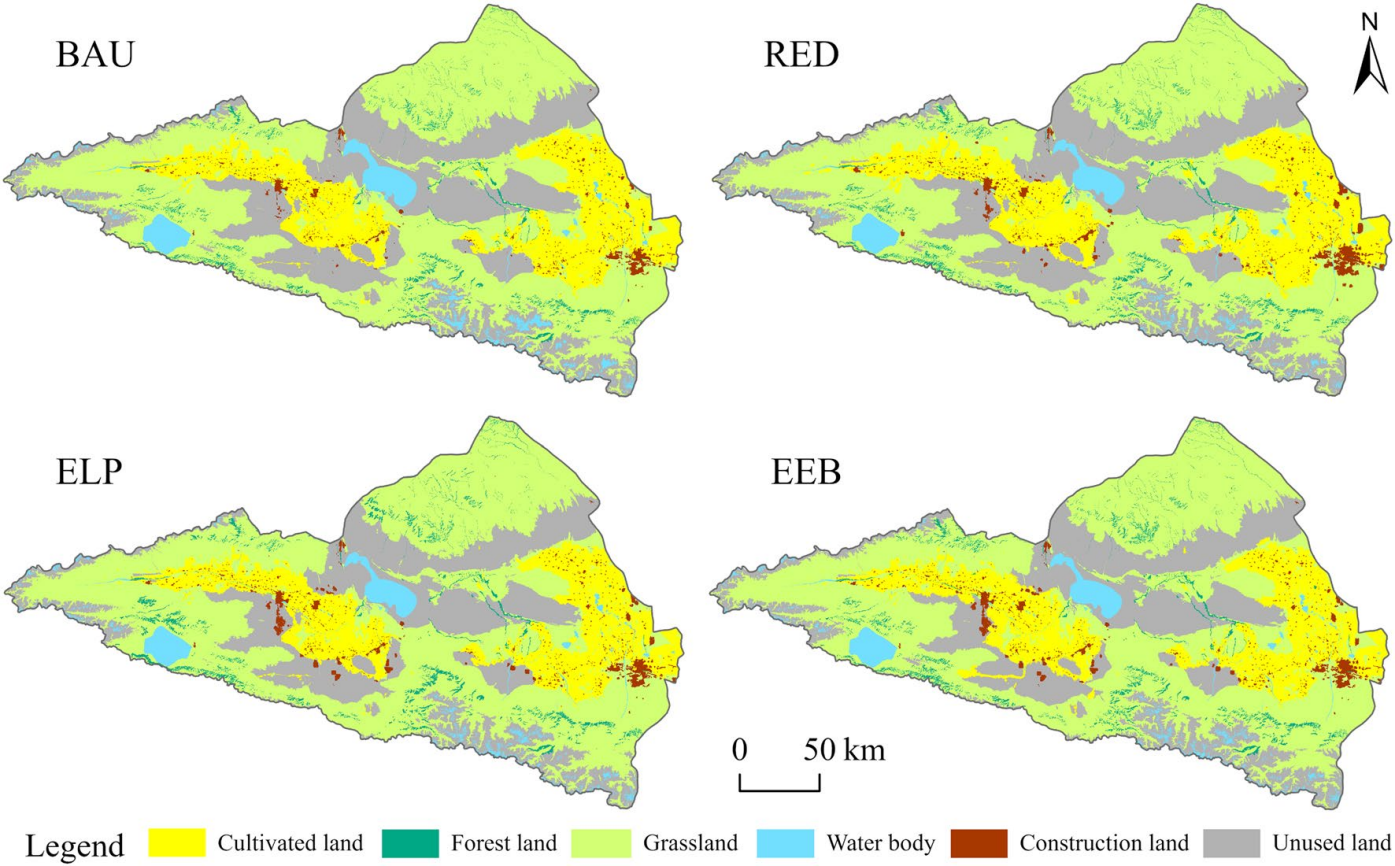
Spatial distribution of LUCC in four scenarios in 2035 (Software: ArcMap 10.2.0, http://www.esri.com).

The coupled GMOP-PLUS model simulated the LUCC in 2035 in the Ebinur Lake Basin under four scenarios (Fig. 8). Compared with 2020, the area of cultivated land generally increased in all four scenarios, with the largest increase observed in the RED scenario, reaching 27.2%. This increase is attributed to the region's population growth driven by socioeconomic development, thereby increasing the demand for food. The growing need for cultivated land directly resulted in the development of unused land. Additionally, due to the government's ongoing comprehensive ecological and environmental management projects, unused land was further transformed into grassland, leading to a continuous reduction in its area. Construction land increased in all scenarios except for a slight decrease in the BAU scenario, influenced by the continuous development of productivity and the rise in urbanization levels. In the RED scenario, which prioritizes economic development, the increase in construction land was most pronounced, reaching 50%. The ELP scenario, which focuses on ecological and environmental protection and sustainable development, shows forest and grassland expansion.

### Characteristics of ESV changes under different scenarios

**Figure 9:**
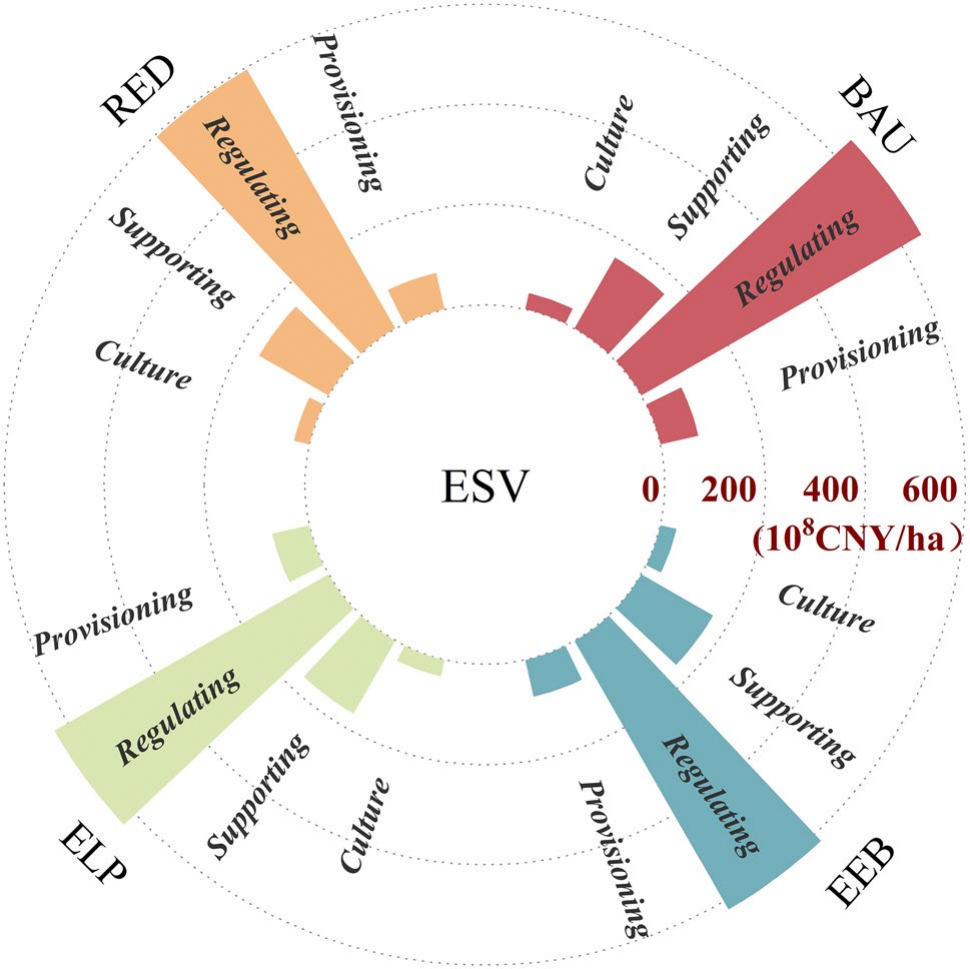
Radial bar chart of ESV for the different functions in the four scenarios in 2035.

Based on the simulated land use changes in 2035, we calculated the ESV under the four different scenarios (Fig. 9). The total ESV in 2035 was 68.83, 64.47, 67.99, and 66.79 billion yuan for the BAU, RED, ELP, and EEB scenarios, respectively. In comparison with 2020, the BAU and ELP scenarios increased by 1.55 and 0.71 billion yuan, respectively, whereas the RED and EEB scenarios decreased by 2.81 and 0.49 billion yuan, respectively. In the ELP scenario, the ESV of all services increased, whereas in the RED scenario, except for a slight increase in provisioning services, all other ESV of services decreased. This is mainly because this study specifies the ESV equivalent factor of construction land per unit area as zero, and the value of each specific service function decreases as a result of other types of LULC being converted into construction. In the BAU scenario, provisioning and regulation services increased by 6.05% and 2.93%, respectively, whereas support and cultural services showed a slight decline. Therefore, the overall ESV increased by 2.3% since 2020. In the EEB scenario, economic development and ecological protection were balanced, land development and utilization were more reasonable, and changes in various service types were relatively small, with a total ESV decrease of 0.73%. In all four scenarios, the increase in cultivated land area significantly enhanced the ESV provided by food production services, resulting in various increases in provisioning services across all scenarios. Due to the high value of individual services provided by water bodies in hydrological regulation and environmental purification, the water body area in the BAU scenario was the most extensive; therefore, the ESV of regulation services showed an upward trend. In contrast, in the RED scenario, where water bodies were reduced to the greatest extent, the ESV provided by regulation services decreased significantly.

**Figure 10:**
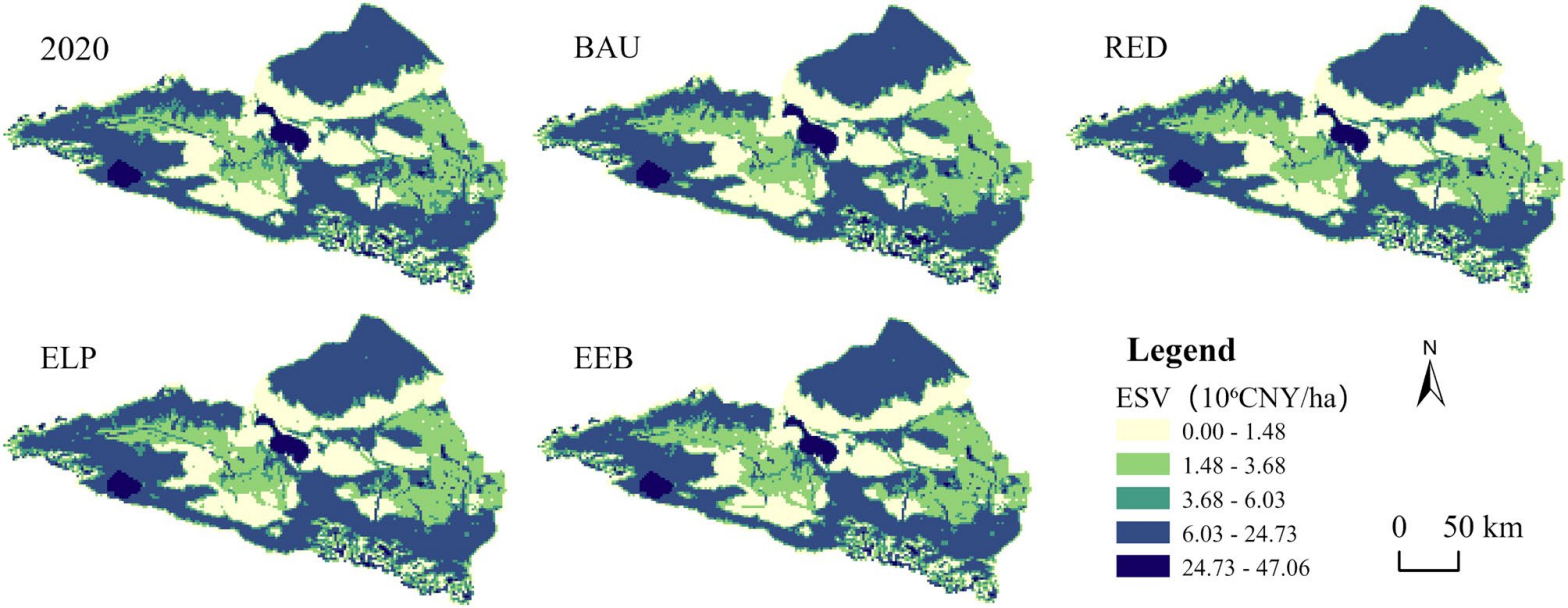
Spatial distribution of ESV in 2020 and under four scenarios in 2035 (Software: ArcMap 10.2.0, http://www.esri.com).

The spatial distribution of the ESV in the Ebinur Lake basin in 2035 under the four scenarios is shown in Fig. 10. The overall distribution pattern was essentially consistent with that in 2020; however, each scenario had local differences. The areas with low ESV were primarily located in the central and eastern parts of the study area, consisting of unused and cultivated lands. The medium- to high-value zones were widespread throughout most of the study area, predominantly comprising grasslands and water bodies. Compared with 2020, ESV declined in areas with expanding construction land in 2035 under all four scenarios. The areas where the ESV increased were mainly located in the southeastern water body distribution area. The majority of other areas in the study region did not change substantially. In the RED scenario, ESV predominantly declined in the study region, whereas in the ELP scenario, the area where unused land is transformed into grassland shows the most obvious increase in ESV. In contrast, the area where the ESV increased was substantially smaller in the EEB scenario than that in the ELP scenario.

## Discussion

### Response of ESV to LUCC

The link between LUCC and ecosystem services is reflected in the differences in a single ESV provided by different LULC, which leads to variations in the overall regional ESV^5^. Half of the study area was grassland and the water body provided the highest single ESV. Therefore, the ESV of the entire study region was primarily influenced by the prevalence of these two dominant cover types.

Between 1990 and 2020, cultivated land, grassland, and construction land showed a noticeably increasing trend. Cultivated land and grassland increased by 355,913 hm^2^ and 95,380 hm^2^, respectively, resulting in an ESV increase of 2.43 and 30.64 billion yuan. As a land use type with excessive human interference, construction land positively affected cultural service functions; however, some of these functions have an adverse effect on ecosystem services (e.g., water pollution and solid waste pollution). Therefore, referring to the unit area ESV equivalent table given by Xie et al.^62^, the ESV provided by construction land in this study was regarded as zero, and any land use type transferred to construction land indicated a net loss in ESV.

The type of land use transformation has a significant impact on how the ESV changes. In the BAU scenario, the direct cause of the rise in ESV is the conversion of unused land into water bodies, with higher unit value equivalents and a conversion area of 28,098 hm^2^. Water bodies contribute significantly to water conservation, environmental purification, and other services. Therefore, this transformation is crucial to the development of ESV in this scenario. In the RED scenario, 213,341 hm^2^ of grassland was transformed into farmland and construction land with lower unit value equivalents. In this scenario, the rise in agricultural irrigation water from the expansion of cultivated land causes a drop in the water area, which is directly linked to a decline in the ESV. In the ELP scenario, the water area remained unchanged from that in 2020, construction land expanded slightly, and cultivated land, forest land, and grassland increased. This was primarily because unused land with a lower ESV was converted. Therefore, the total ESV of the ELP scenario increased significantly compared with that in 2020. Although woodland and grassland areas decreased in the ELP scenario, both were larger than that in the BAU and RED scenarios.

Overall, the ELP scenario balances economic benefits and environmental protection, and improves various service functions compared with the RED scenario. However, such conclusions are not consistent in terms of the distribution of ESV in specific regions. For example, in the southeastern mountainous region of the study area, water bodies in the RED scenario were larger than that in the ELP scenario because of the snow and ice cover; therefore, the ESV of this region in the RED scenario was higher than that in the ELP scenario. It can be seen that the same scenario cannot achieve the optimal land use strategy in the Ebinur Lake Basin, and the optimization of LULC should not be limited to a single scenario. Instead, different strategies should be adopted according to the characteristics of the region to achieve sustainable development.

### Land policy planning based on ESV evolution

This study evaluated the temporal progression of the ESV from 1990 to 2020 and predicted future ESV under four different scenarios. These findings may serve as a reference for the government to develop high-quality spatial development plans for national land. When comparing the optimized land use plans under the ELP, EEB, BAU, and RED scenarios, ELP and EEB exhibited a higher degree of sustainability. The ELP scenario demonstrates a high degree of suitability for ecological conservation, limiting the large-scale occupation of grasslands for the reclamation of cultivated land. The EEB scenario balances the economy and ecology, and a reasonable land use pattern is conducive to bringing higher economic benefits to a healthy ecosystem. Hence, decision-makers should carefully consider the carrying capacity of the environment and develop safe and sustainable spatial land-use plans. Based on our findings, we recommend that the following specific measures be implemented:

In the central and eastern cultivated areas of the study area, permanent basic farmland must be delineated and positive optimization should be conducted. Furthermore, protection around prime agricultural areas must be reinforced and the disorderly expansion of cultivated land must be avoided. The growth in agricultural land acreage has resulted in more water being used for irrigation, which has decreased the water area within the study region. Therefore, enhancing the ability of “storage and allocation” and improving irrigation methods is conducive to protecting the water area and increasing the contribution of water bodies to the ESV. In grassland and woodland ranges, the ecological protection red line should be delineated in accordance with ecological functions, forest and grassland resources must be protected through zoning, and differential control should be achieved. Unused land should be reasonably developed to promote the stable development of forest and grassland ecosystems with high ESV. In areas where construction land is concentrated, the urban development border must be defined in accordance with the principles of intensive and moderate green development. Additionally, global tourism development should be supported in places such as Sayram Lake and Wenquan County, and the cultural service function of construction land should be improved.

### Causes of sustained ESV increase

**Figure 11:**
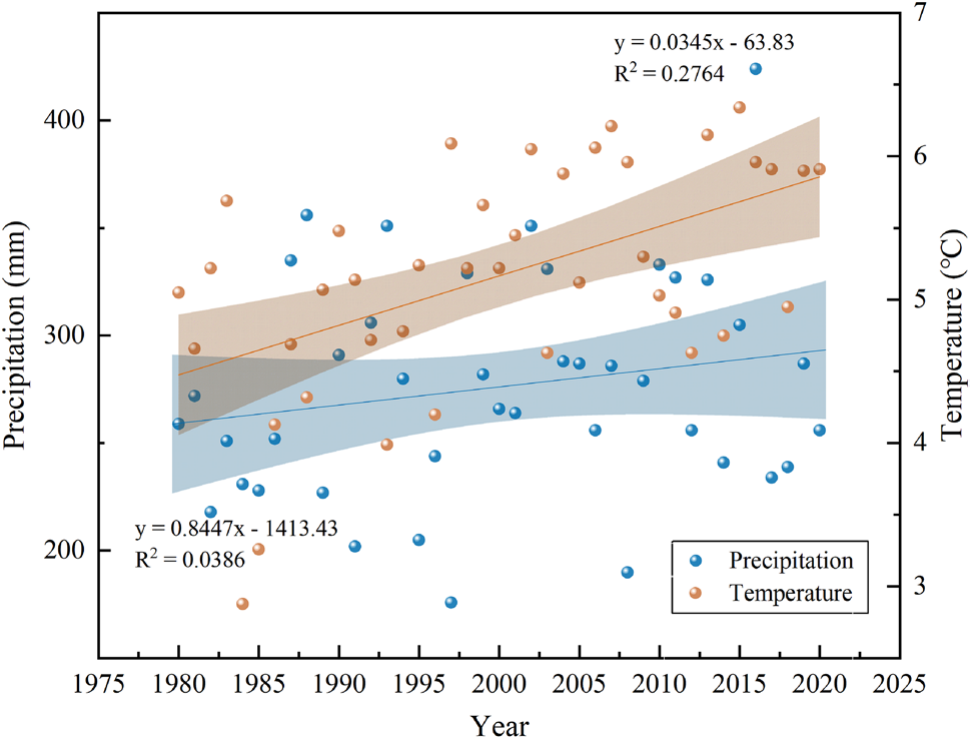
Trends in precipitation and temperature in the Ebinur Lake Basin from 1980 to 2020.

The total ESV and several ecological service functions in the study region showed consistently increasing trends from 1990 to 2020. Forestland and water bodies persistently shrank but still contributed to the increase in ESV. To explore the causes, the following points are primarily considered:

1. The continued increase in planting area, yield, and price of food crops contributed to the increase in the standard equivalents of ESV in 1990, 2000, 2010, and 2020.
2. After the spatial heterogeneity correction of ESV by NPP and FVC, the biomass factor coefficient continued to increase. Zhao et al.^63^ showed that since 1982, the NDVI in Xinjiang has been increasing, and this can sufficiently explain the rise in ESV, which is consistent with the conclusion that the biomass factor coefficient shows an upward trend. This demonstrated the success of the government's efforts to restore and safeguard the ecological environment.
3. With the advancement of society and the economy and changes in people’s ideas, people’s ability and willingness-to-pay for ecological services have increased, which has resulted in a considerable rise in ESV, which aligns with previous findings^64,65^.
4. The accurate establishment of the equivalent factor is key for evaluating the ESV. The growth of the social economy and changes in individuals' cognitive concepts significantly impact ESV. For 1990, 2000, and 2010, we used the value-equivalent table proposed in 2003^62^. In 2015, the unit area value equivalent factor method improved and developed by Xie et al.^65^ added two service types: water supply and nutrient cycle maintenance. This improved value equivalence table was subsequently used in 2020, which greatly increased the contribution of grasslands and water bodies to the ESV. This explains why the total ESV of 1990–2010 increased by approximately 30%, whereas the total ESV of 2010–2020 increased by up to 110%.
5. During the evaluation period, unused land with a low unit value continued to shrink, with its area ratio decreasing from 34.35% to 27.6%, and transformed into grassland and farmland ecosystems with higher unit values. This is one reason for the continuous ESV increase.
6. Warming and humidifying trends positively influenced the ESV increase. Figure 11 illustrates the fluctuating upward trends in temperature and precipitation in the Ebinur Lake Basin over the last 40 years. Climate warming and humidification augment the areas of forest and grassland, and the NDVI value also increase, thus promoting an increase in ESV. This aligns with the findings of previous studies conducted on the wetlands in Ebinur Lake^49^.

Drawing from the above reasons, the ESV within the study region increased continuously from 1990 to 2020. This trend is consistent with the results of previous studies conducted in the Xinjiang region^29,66,67^.

### Advantages, limitations, and future research

Previous studies^14,24,68^ mostly used the average value of the evaluation year to establish a standard equivalent, ignoring the spatiotemporal dynamic evaluation of the ESV. In this study, we coupled the GM (1,1) grey linear prediction model, fully considering the impact of the biomass factor, payment ability, and willingness-to-pay on the ESV equivalent factor, and constructed unit equivalent factor tables for 1990, 2000, 2010, and 2020. In 2020, the equivalence table improved in 2015 was used because a developed equivalent value improves the credibility of evaluation results^65^. Therefore, this study provides an objective and accurate dynamic ESV evaluation model.

Despite such advantages, some limitations remain. In the scenario simulation, we did not fully consider the effects of human activities and climate change. Future research should combine shared socioeconomic pathways and representative concentration pathways with the GMOP to develop more reasonable scenario settings. Furthermore, this study did not consider the ESV of construction land. In the future, we should comprehensively consider the aesthetic and cultural services provided by construction land as well as its adverse effects on the environment to establish a more comprehensive and accurate evaluation model. In addition, this study did not consider the supply–demand matching of various services, as well as the trade-off and synergy among them, which is a crucial area for future study.

## Conclusions

This study used a coupled GMOP-PLUS model and the equivalent coefficient method to accurately evaluate LUCC patterns and ESV changes in the Ebinur Lake Basin from 1990 to 2020 and predict four scenarios in 2035. The results show that from 1990 to 2020, the Ebinur Lake basin was primarily characterized by grasslands and unused land, with unused land subsequently transforming into grasslands and farmlands. Over 30 years, the total ESV increased from 18.62 billion to 67.28 billion yuan. Grasslands and water bodies contributed significantly to the increase in ESV, with regulation and support services being the dominant functions in the study area. By 2035, the overall distribution pattern of ESV is expected to remain largely consistent with that of 2020, with an increase in cultivated land area under all four scenarios and a decrease in unused land. The most notable increase in construction land is projected under the RED scenario, reaching 50%. Under the ELP scenario, ESV is expected to increase for all services. This study reveals that the structural and pattern changes in LULC significantly impact ESV, and the four future scenarios can provide decision-making references for land resource planning and ecosystem management.

## Data Availability

The datasets used in this study are accessible in the *OSFHOME* repository, https://osf.io/hjuam/?view_only=ca69a5f5bcf04f1a9ad849ecfb5dfa77. The *China Statistical Yearbook, Xinjiang Statistical Yearbook, Bortala Mongol Autonomous Prefecture Statistical Yearbook* datasets used during the current study available from the corresponding author on reasonable request.
